# Supplementary material for: On the Stability of Electrohydrodynamic Jet Printing Using Poly(ethylene oxide) Solvent-Based Inks
Source: Nanomaterials (Basel). 2024 Jan 27;14(3):273. doi: 10.3390/nano14030273 (PMC10856662; doi:10.3390/nano14030273)
Supplement: Supplementary file 1 [file nanomaterials-14-00273-s001.zip › nanomaterials-2828429-supplementary.pdf]

Supporting Information

On the stability of electrohydrodynamic jet printing using poly(ethylene oxide) solvent-based inks

Alberto Ramon[1], Ievgenii Liashenko[1,2,3], Joan Rosell Llompart[2,4,*], Andreu Cabot[1,4,*]

[1] *Catalonia Institute for Energy Research (IREC), Jardins de les Dones de Negre 1, 08930 Sant Adrià de Besòs, Barcelona, Spain*

[2] *Department of Chemical Engineering, Universitat Rovira i Virgili, Av. dels Països Catalans 26, 43007 Tarragona, Spain*

[3] *Phil and Penny Knight Campus for Accelerating Scientific Impact at University of Oregon, 1505 Franklin Boulevard, Eugene, 97403 OR, USA*

[4] *Catalan Institution for Research and Advanced Studies (ICREA), Pg. Lluís Companys 23, 08010 Barcelona, Spain*

* joan.rosell@urv.cat, acabot@irec.cat

Contents

Figure S1 3

Figure S2 4

Figure S3 5

Figure S4 6

Figure S5 7

Figure S6 9

Table S1 10

Table S2 11

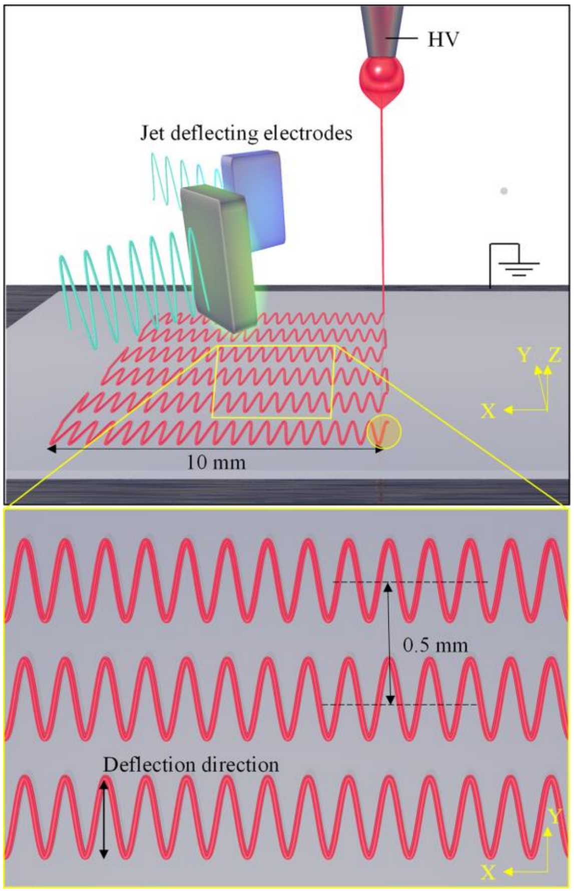

Figure S1. Schematics of the electrostatic jet deflection printing and travel of the jet (default trajectory) over the substrate. Continuously, during printing, the jet is periodically deflected by auxiliary electrodes along the Y direction. Initially, the mechanical stage translates along the X-axis for 10 mm from an initial X=0 position (orange circle), then stops at X=10 mm and moves +0.5 mm along the Y-axis (without interrupting the jetting). After that, the stage moves again along the X-axis, but in the reverse direction, from X=10 mm back to X=0. After a new Y-axis displacement of +0.5 mm, this cycle is repeated until a 10 mm x 10 mm square has been printed, containing 20 long tracks parallel to the X-axis.

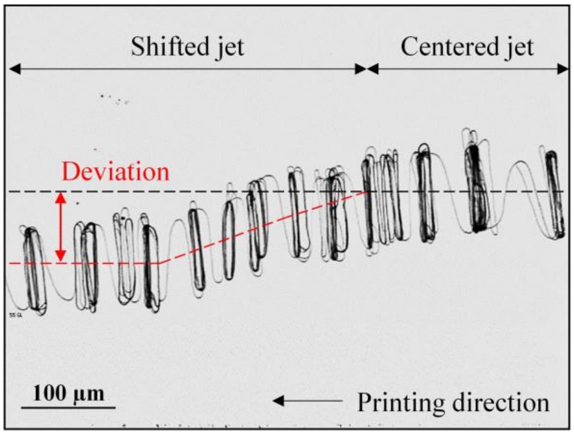

Figure S2. Jet deviation measurement. Confocal image of a printed fiber and schematized methodology used for measuring the deviation of the jet after the printing. The ink in this example is composed of 3 wt% 1 MDa PEO dissolved in H_2O:EtOH (1:1 wt), and the nozzle voltage and the infusion pump rate were 850 V and 30 nL min^{-1}, respectively. To quantitatively measure the jet deviation, first, a theoretical default jet trajectory was specified. Its starting point was placed at the location where the jet was initially ejected to start the experiment. Then, the jet's "default trajectory" was defined as that followed by the stage pathway from the starting point (dotted black line), which was the set of zigzag movements described in Figure S1. The deviation from the jet's default trajectory was determined as the distance (positive or negative) between the fiber track centerline (red dotted line) and the default jet trajectory (dotted black line).

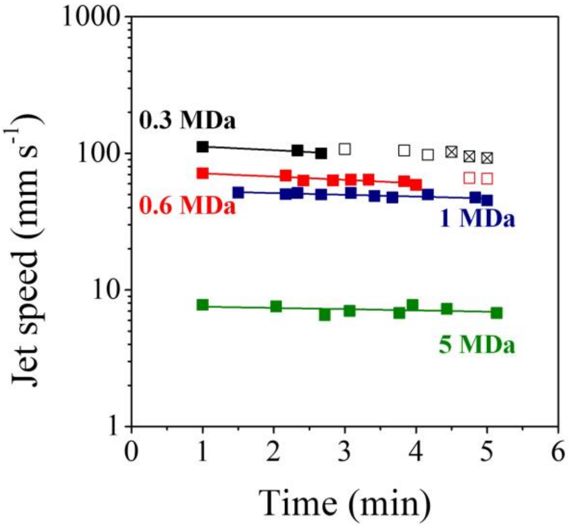

Figure S3. Effect of the polymer molecular weight on the jet speed stability at constant zero-shear viscosity; same data shown in Figure 3 with the ordinate in logarithmic scale. Needle voltage was 750 V, while infusion pump rate was 70 nL min^{-1} (Table S2). At time 0, the jet was initiated. For the 0.3 and 0.6 MDa sets, the different symbol fillings represent data obtained from different initiations of the jet after it spontaneously had become interrupted. Solid lines show the linear fitting of the time-dependence of the jet speed before its interruption.

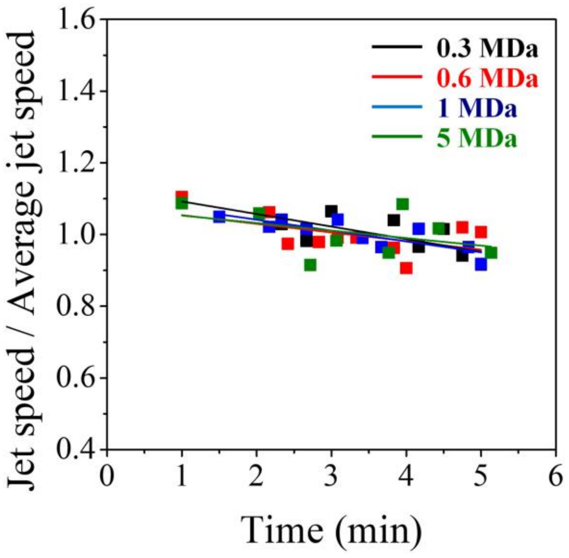

Figure S4. Normalized jet speed over time while using different PEO molecular weight (from Figure S3). The normalized jet speed decay was similar in all PEO molecular weights, but it was a bit more evident (faster) for the lowest molecular weight.

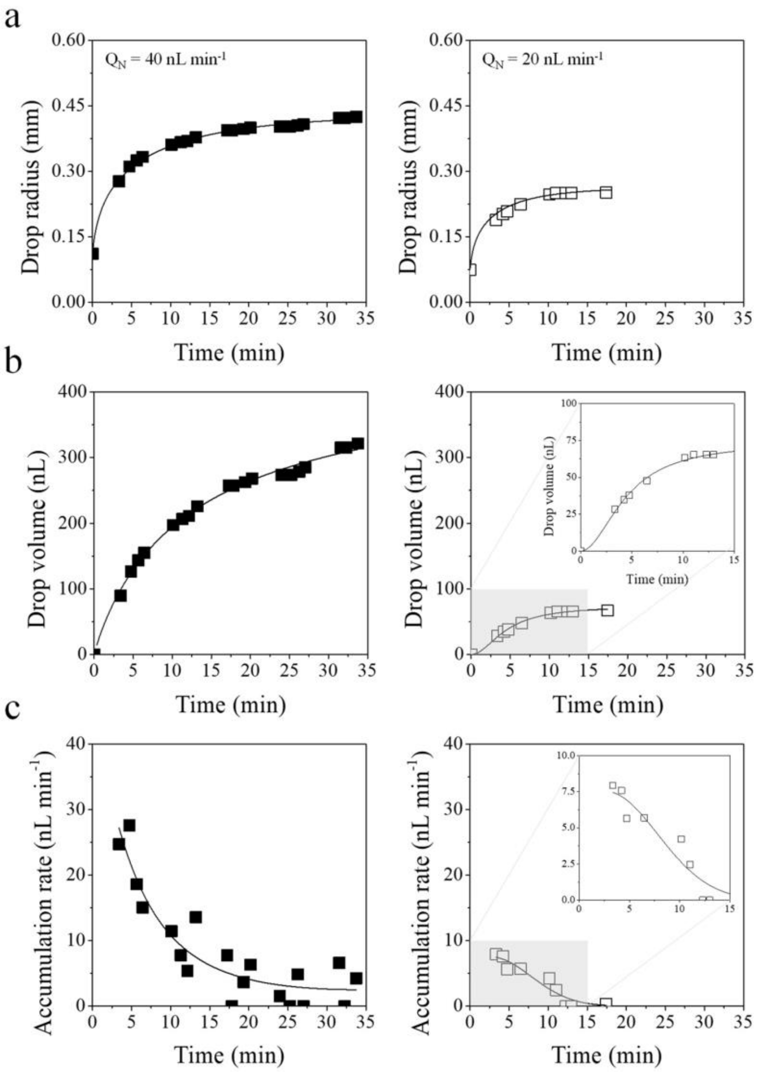

Figure S5. Drop size evolution at different infusion pump rates. The ink consists of 3 wt% PEO 1 MDa dissolved in H_2O:EtOH (1:1 wt), and the nozzle voltage was set at 1000 V. (a,b) Evolution of the drop size (a) and its volume (b) during the printing process the infusion pump rate was set at 40 nL min^{-1} (left graphs) and 20 nL min^{-1} (right graphs). Fitting lines in (a) are based on equation (10), while experimental data points in (c) were adjusted using “LangmuirEXT1” function from Origin (for 40 nL min^{-1}: a=438.6, b=0.0931, c=0.0678; for 20 nL min^{-1} a=74.0, b=0.054, c=0.950). (c) Estimated ink accumulation rate obtained from the derivative of the drop volume data. In panels b-c for the 20 nL min^{-1} case, insets show a magnified view of the data at short times.

Figure S5 shows that the drop was continuously growing for more than 30 minutes when a relatively high infusion pump rate (40 nL min^{-1}) was used. During this time, the jet speed kept similar within a range of 30 – 40 mm s^{-1} (Figure 4). In contrast, when a much lower infusion pump rate was set (20 nL min^{-1}), the drop surface dried and the printing time could not be longer than 15 minutes. The magnified graphs shown as insets in Figure S5(b,c) display that the drop was growing until it reached a maximum volume. At that moment, the total volume of ink that was supplied to the drop (infusion pump rate) was similar to the total volume of ink that left the drop (jet and evaporated solvent), so the drop volume stopped increasing and its surface started to dry up. This hypothesis is supported by the data in Figure 4 of the fiber diameter evolution over time, which increased during the printing.

Using a relatively high infusion pump rate allows minimizing the drying of the drop surface, as new fresh ink enables diluting the concentrated polymer, so variations on the drop properties and jet speed are reduced (Figure 4, data set for 40 nL min^{-1}). However, this situation does not assure printing in steady state, as using too high infusion pump rates implies having drops with huge variations in size, which would perturb the electrical field around the jet.

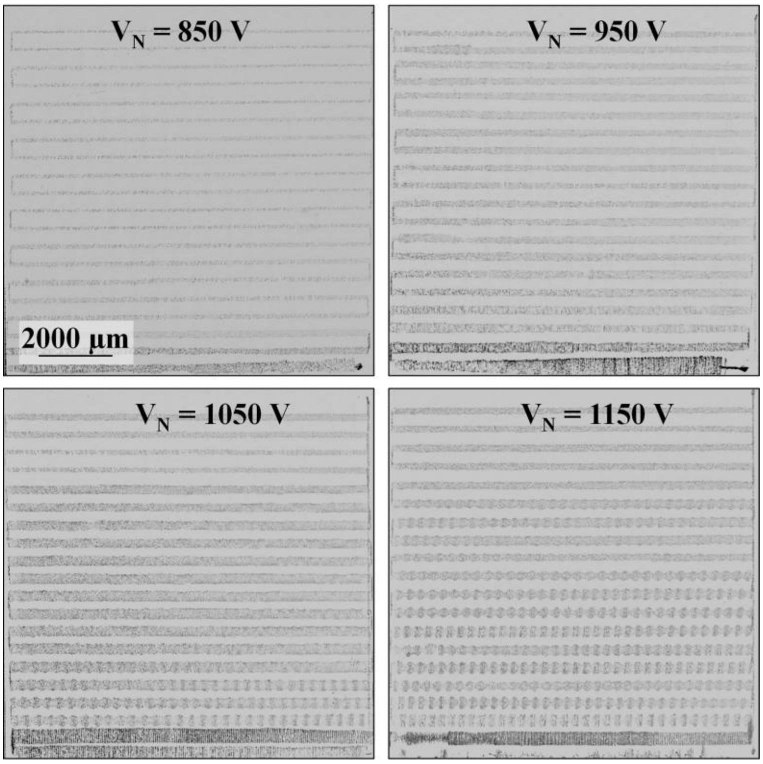

Figure S6. Effect of the nozzle voltage on the jet ejection mode. These confocal microscopy images display the whole printed area showed in Figure 5. The ink used is composed of 3 wt% 1 MDa PEO dissolved in H_2O:EtOH (1:1 wt). Infusion flow rate was fixed at 20 nL/min, and nozzle voltage was varied from 850 V to 1150 V. Each confocal microscope image shows independent experiments that started after cleaning the nozzle to get a fresh drop. Then, the jet was initiated at a specific nozzle voltage and it was ejected for 2 minutes for stabilization, while the substrate was moved slower (see Methods), resulting in the denser two lines at the bottom of each panel.

Table S1. Ink compositions used in this study and their properties: PEO's of different viscosity-average molecular weights (M_v) were dissolved at different concentrations in a solvent composed of a H_2O:EtOH mixture (1:1 by wt) to analyze the effect of the polymer molecular weight on the jet speed and its stability. Additionally, 1 MDa PEO was dissolved in a H_2O:EG mixture (4:1 by wt) to examine the effect of the drying of the drop surface on the jet ejection point. n_e indicates the entanglement number of each ink. Before the measurement of the ink properties, the inks were kept inside an oven at 25ºC overnight, and then the zero-shear viscosity (η_0) and electrical conductivity (σ) measurements were carried out at room temperature.

**Ink batch**	**PEO M_v (MDa)**	**PEO wt %**	**Solvent**	n_e *	**η_0 (Pa·s)**	**σ (µS/cm)**
#1	0.3	5.5	H_2O:EtOH (1:1, wt)	6.1	1.92	13.1
#2	0.6	3.9		8.5	1.87	11.4
#3	1	3		10.7	1.84	9.0
#4	5	1.4		24.6	1.90	6.1
#5	1	3	H_2O:EG (4:1, wt)	12.4	4.22	39.2

* The entanglement number n_e was estimated using equation S1 (Suresh L. Shenoy, W. Douglas Bates, Harry L. Frisch, Gary E. Wnek. Role of chain entanglements on fiber formation during electrospinning of polymer solutions: good solvent, non-specific polymer–polymer interaction limit. Polymer (2005). Volume 46, Pages 3372-3384):

$$n_e = \frac{\phi_p \cdot M_w}{M_e} \quad [S1]$$

where ϕ_p is the polymer volume fraction, M_w is the polymer weight-average molecular weight and M_e is the entanglement molecular weight.

Table S2. Experimental conditions used in the figures shown in the supporting information file**.** Ink composition, operational parameters (i.e. infusion pump rate, Q_L; amplitude and frequency of the jet deflecting signal, A and ν, respectively; and nozzle voltage, V_N) and ambient conditions (temperature, T and relative humidity, RH) of the experiments.

**Figure**	**Ink***	Q_L **[nL min^{-1}]**	A **/** ν **[V / Hz]**	T **/** RH **[°C / %]**	V_N **[V]**
S2	3 wt% 1MDa-PEO H_2O:EtOH	30	2000 / 500	18 / 60	850
S3	5.5 wt% 0.3MDa-PEO H_2O:EtOH<br>3.9 wt% 0.6MDa-PEO H_2O:EtOH<br>3 wt% 1MDa-PEO H_2O:EtOH<br>1.4 wt% 5MDa-PEO H_2O:EtOH	70	2000 / 300<br>2000 / 250<br>2000 / 150<br>2000 / 50	18/40	750
S4	3 wt% 1MDa-PEO H_2O:EtOH	20, 40	2000 / 200, 300, 400	18 / 50	1000
S5	3 wt% 1MDa-PEO H_2O:EtOH	30	2000 / 350	18 / 50	950

* Note: The H_2O:EtOH and H_2O:EG solvent mixtures were 1:1 and 4:1 by wt, respectively. The nozzle to collector distance and stage translation speed were set at 3 mm and 1 mm s^{-1}, respectively.
